# Dental Hospitals

**Published:** 1862-05

**Authors:** 


### DENTAL HOSPITALS.

The following from the Dental Review, London, contains some good suggestions, and presents several important points, in reference to the practicability and usefulness of Dental Hospitals.

We give the article entire, and suggest for it the careful consideration of every reader of the Register.—Ed. Reg.

Numerous as are the ills which flesh is heir to, few are so universal as diseases of the teeth. From the period of boyhood until the time comes when man passes away like a shadow, the teeth too often remind him of the frailty of his body. Although the diseases themselves are not dangerous to life, yet they may gradually undermine the health, and lay the foundation of premature decay; what they want in importance, they make up in frequency.

Of the several specialties into which surgery has become divided, dentistry was one of the earliest, and the one most generally recognized. Like every other branch of practice, it has of late years been greatly improved, both as regards its scientific character, and the estimation in which it is held by the public. The time is within the recollection of some who are still in practice, when a visit to the dentist was performed with the utmost secresy. The fashionable lady left her carriage a few streets off, lest John, the footman, should proclaim where his mistress had alighted. Let it only be known that the beauty of the season, whose name had been the reigning toast, had consulted the Dentist, and her charms vanished, as if under the spell of some malignant enchanter. A call upon the popular physician excited the commiseration of sympathizing friends; but to be tracked to the door of the dentist, was followed by shrugs of the shoulders, and whispered inuendoes that you had not a tooth of your own. The common sense of mankind has happily done away with these absurd notions. It is known that the teeth will decay, that they are essential not only to the personal appearance, but also to the health of the individual, and a visit to the dentist is no longer regarded as something to be ashamed of.

While, however, these changes have taken place amongst the higher and more wealthy classes of society, what in such matters has been the fate of the poor and needy? Those who gain their bread by the sweat of their brows seldom pay any attention to their teeth until they become a source of suffering or annoyance. Such persons are neither of a temperament, nor in a position to put up for long with that "hell o' a disease," the tooth-ache. When a man's subsistence depends upon each day's labor, the loss of two days through illness or incapacity disarranges his finances for a month to come. The only resource of this important class of the community in these diseases has hitherto been the general hospitals, and the only relief the latter afforded was the extraction of the offending organ. The walls of these noble foundations are only crowded with those who are suffering from mortal diseases, or who have been overtaken by some terrible accident. The pallid, miserable, and emaciated infant, telling a tale of poverty and want; the young girl with the fatal hectic upon her cheek; the once stalwart individual, whose occupation has paralyzed his limbs, and reduced him to an incapable and stricken man, the debauched and drunken artizan, none the less an object of compassion, that his own folly has worked his ruin,—these, and many other cases equally melancholy, and equally helpless, claim the attention of the hospital surgeon, and find employment for the funds that charity dispenses with no niggard hand within the precincts of our vast metropolis. Should the poor man visit the general dispensaries, he is no better off as regards his teeth than he was at the hospital, since the same causes militate against the proper treatment of their diseases. More important cases establish a stronger claim upon the funds of the institution and the attention of the medical officers. In many dispensaries, it is true that of late years dentists have been appointed to take charge of all cases relating to the teeth. Notwithstanding these appointments, in consequence of the want of funds, and the absence of a proper organization, the treatment of the teeth has still, with rare exceptions, been limited to the operation of extraction.

The only remedy for this state of things was to provide special hospitals or dispensaries, exclusively devoted to the treatment of diseases of the teeth. Two such hospitals are now established in London. Amongst the advertisements of this journal will be found a list of the patrons, officers, and subscribers of the youngest, but apparently not the least vigorous of the two establishments. The want of such institutions can hardly be questioned, when we witness the ready support they have received from the charitably disposed, and know the number of applicants who daily present themselves for relief. The time must come when all the large towns will be provided with dental hospitals; but until this takes place, those of London have claims upon the support, not only of the public, but the profession itself. It is, we conceive, the duty of every dentist, who has the good of his profession at heart, to aid, as far as lies in his power, in securing the prosperity of these charities. We are glad, therefore, to find, from examining the list of the supporters of the National Dental Hospital, the one before us, that this claim upon the profession has been recognized, not only by the dentists of the metropolis, but also by some of those who are practising in the provinces. We sincerely trust that the dentists who reside out of London will not allow themselves to be governed by any narrow or selfish views; they may rest assured that ultimately they will reap the full benefit of any assistance they may render to the Metropolitan Dental Hospitals. The general hospitals of the metropolis existed long before the establishment of the provincial institutions of the same kind. In like manner, when the necessity and usefulness of the London Dental Hospitals have been impressed upon the public mind, the way will be prepared for their extension to other parts of the kingdom.

No new institutions of this kind can expect to be received with universal approbation; with some persons every innovation is a dangerous revolution, and every change a deviation from a good old custom. Some are filled with groundless alarms lest dental hospitals should be used improperly, and exercise an injurious influence over a certain class of dental practitioners. That in some few cases these charitable institutions will be abused, there can be little doubt. There are men so wedded to gold that, like old Elwes, the miser, they would sooner submit to have two sound teeth extracted, than part with their money for nothing. Others are so imbued with the spirit of fraud, that they will spend five shillings to evade the honest payment of half-a-crown. Are, however, these perversions of human nature to prevent us from benefitting a large body of our fellow creatures? and is the hand of charity to be closed because mean and sordid minds exist in the world?

It is the bounden duty of the officers of the dental hospitals, as well as of all others, to take care that they are not used by persons for whom they are not intended. It is the province of the committee of management to see that the funds are not misapplied, while those who act as auditors of the annual accounts should in like manner be a check against any laxity on the part of the committee. With these safeguards against the abuse of the dental hospitals, we have no fear of their ultimate success, while we are fully convinced of the great boon they will prove to the poorer classes. Inasmuch, however, as dental hospitals are new institutions, questions will necessarily arise regarding their use and management, which will require careful consideration. While, on the one hand, it is the duty of the dental officers and committee of management to guard against encroachment, so, on the other hand, it is equally their duty to take care that the poor, for whose use these charities are intended, should receive all the benefits they are capable of affording. There are points connected with the management of dental hospitals which we may refer to on some future occasion. At present, our object is to bring these establishments under the notice of the profession, and to express a hope that they will receive all the support we feel they deserve.

